# Urinary Calcium and Oxalate Excretion in Healthy Adult Cats Are Not Affected by Increasing Dietary Levels of Bone Meal in a Canned Diet

**DOI:** 10.1371/journal.pone.0070530

**Published:** 2013-08-05

**Authors:** Nadine Passlack, Jürgen Zentek

**Affiliations:** Institute of Animal Nutrition, Department of Veterinary Medicine, Freie Universität Berlin, Berlin, Germany; Auburn University, United States of America

## Abstract

This study aimed to investigate the impact of dietary calcium (Ca) and phosphorus (P), derived from bone meal, on the feline urine composition and the urinary pH, allowing a risk assessment for the formation of calcium oxalate (CaOx) uroliths in cats. Eight healthy adult cats received 3 canned diets, containing 12.2 (A), 18.5 (B) and 27.0 g Ca/kg dry matter (C) and 16.1 (A), 17.6 (B) and 21.1 g P/kg dry matter (C). Each diet was fed over 17 days. After a 7 dayś adaptation period, urine and faeces were collected over 2×4 days (with a two-day rest between), and blood samples were taken. Urinary and faecal minerals, urinary oxalate (Ox), the urinary pH and the concentrations of serum Ca, phosphate and parathyroid hormone (PTH) were analyzed. Moreover, the urine was microscopically examined for CaOx uroliths. The results demonstrated that increasing levels of dietary Ca led to decreased serum PTH and Ca and increased faecal Ca and P concentrations, but did not affect the urinary Ca or Ox concentrations or the urinary fasting pH. The urinary postprandial pH slightly increased when the diet C was compared to the diet B. No CaOx crystals were detected in the urine of the cats. In conclusion, urinary Ca excretion in cats seems to be widely independent of the dietary Ca levels when Ca is added as bone meal to a typical canned diet, implicating that raw materials with higher contents of bones are of subordinate importance as risk factors for the formation of urinary CaOx crystals.

## Introduction

The role of dietary calcium (Ca) and phosphorus (P) in the formation of feline uroliths is subject of controversy. It has been suggested that restricting dietary Ca would reduce the renal Ca excretion and therefore lower the risk for Ca oxalate (CaOx) uroliths in cats [1]. Interestingly, studies in humans and dogs indicate that higher concentrations of dietary Ca may cause an intestinal complexation of Ca with oxalate (Ox), resulting in reduced concentrations of urinary Ca and Ox [2]–[3]. The amount of dietary oxalate in diets for cats is normally low, and therefore no remarkable intestinal complexation with dietary Ca can be expected in this species. Additionally, the renal Ca excretion in healthy cats is small and seems not strictly correlated with the dietary Ca intake [4].

Beside its relevance for the formation of CaOx uroliths, dietary Ca may be a potential risk factor for the development of magnesium ammonium phosphate (MAP) urolithiasis in cats, mainly depending on its effects on the acid base metabolism [5] and on blood Ca concentrations. Physiologically, hypercalcaemia from high intestinal Ca absorption results in a reduced secretion of parathyroid hormone (PTH). The reduced levels of PTH may inhibit the renal tubular reabsorption of magnesium (Mg) and enhance the urinary supersaturation with Mg, ammonium and phosphate [6], increasing the risk of MAP formation. In addition, high dietary Ca levels are often accompanied by high P concentrations in the diets to maintain an adequate Ca:P-ratio. High dietary P levels lead to an increased intestinal absorption of P and enhanced renal P excretion, which is considered as another predisposing factor for the formation of MAP [1]. High urinary Ca concentrations can also contribute to the formation of Ca phosphate uroliths [7], although they are by far less common compared to CaOx or MAP [8].

The dietary Ca salts have a major impact on the nutrient and acid base metabolism in cats that must not be neglected when considering potential risks for the formation of uroliths. Increasing dietary Ca chloride and carbonate resulted in a reduced renal excretion of P and Mg [9]. Moreover, the urinary P concentrations were lower when the cats were fed a diet with Ca chloride compared to Ca carbonate. Beside the effects on urinary mineral concentrations, the impact of different Ca and P sources on the acid base metabolism and the urinary pH is a critical factor for the formation of crystals and uroliths. While an acidification of the urine is considered to prevent MAP [10]–[11], a low urinary pH has been identified as a risk factor for the formation of CaOx [1], [12]–[13]. In this context, the salt of dietary Ca has to be considered. Ca carbonate is assumed to be an alkalizing mineral salt [14]–[16], whereas Ca chloride acidifies the urine [17]–[18]. By now, the role of Ca phosphates on the urine composition is unclear, although they are of increasing practical importance because of an intensive use of animal by-products, such as poultry carcasses, in petfood. The use of raw materials with higher levels of bones resp. bone meal in commercial diets results in high dietary Ca levels, but also in high dietary P concentrations. Bones are containing Ca as hydroxyapatite [Ca_10_(PO_4_)_6_(OH)_2_], the crystallized form of Ca phosphate [19]–[20]. As yet, there are no data available for the effects of bone meal resp. Ca hydroxyapatite in cats. Therefore, it was the aim of the present study to investigate the influence of increasing levels of dietary Ca and P, added to the diets as bone meal, on the mineral metabolism in cats. Considering the uncertainty in respect to its role in the formation in feline uroliths, the influence of dietary bone meal on the urine composition was particularly evaluated. The investigations further included measurements of the faecal mineral excretion, the mineral status in the blood, and also the PTH secretion as an important marker for regulatory processes of the Ca metabolism.

## Materials and Methods

### Animal Study

The experimental protocol was approved by the Animal Welfare Committee (Landesamt für Gesundheit und Soziales, Berlin, Germany, G 0421/09). Eight adult cats (European shorthair, 4 male, 4 female, 36±4 months) were included in the present study. At the beginning of the study, the body weight (BW) of the cats was 4.43±1.30 kg, and at the end of the study 4.35±1.18 kg. The cats were housed as a group in a room with a constant light (12 h light/12 h darkness) and temperature (21°C) regime. The cats were fed individually twice a day (7.00 h and 12.00 h), and the daily feed intake was documented for each cat.

The experimental diets were fed over 17 days each. After an adaptation period of 7 days, the cats were housed in metabolic cages during a collection period of 2×4 days (with a two-day rest between). The metabolic cages contained purpose-built cat litter boxes with plastic pellets as litter. The urine of the cats could flow into a connected urine collection container, which was provided with three drops of chlorhexidine-digluconate to prevent a bacterial growth in the urine. The total urine and faeces were collected twice a day and stored at −40°C. On the last day of each feeding period, blood of the cats was collected in the morning when the cats were fasting.

### Diets

Before the beginning of the study, the cats were fed a commercial, dry extruded diet (Table S1).

For the present study, three experimental diets were offered in three feeding periods. The Ca concentrations were 12.2, 18.5 and 27.0 g/kg dry matter (DM) and the P concentrations 16.1, 17.6 and 21.1 g/kg DM (Table 1). The diets based on meat and animal by-products (60%), 15% poultry carcasses (Ca:P-ratio = 1.2∶0.7), chicken bone paste (diet A: 2.5%; diet B: 5.5%; diet C: 9.5%), 0.35% minerals and vitamins, and <1.0% gelling and thickening agent (residual: process water). The diets were formulated to fulfill the recommendations for adult cats [21].

**Table 1:** Nutrient analysis of the experimental diets.

		Diet		
Analyzed composition		A	B	C
Dry matter	g/kg	163	174	174
Crude protein	g/kg DM	607	602	565
Crude fat	g/kg DM	274	267	245
Crude fiber	g/kg DM	2.29	2.72	3.26
Crude ash	g/kg DM	94.5	103	138
Ca	g/kg DM	12.2	18.5	27.0
P	g/kg DM	16.1	17.6	21.1
Ca:P-ratio		0.76∶ 1	1.05∶ 1	1.28∶ 1
Na	g/kg DM	9.72	9.69	10.7
K	g/kg DM	11.5	10.3	10.5
Mg	g/kg DM	0.90	0.93	1.12
Ox	g/kg DM	0.08	0.14	0.06
Metabolizable energy^1^	MJ/kg DM	20.6	20.3	19.2

1 Calculated (NRC 2006).

### pH Measurements and Preparation of the Urine

The urinary pH was measured twice a day at 7.00 h (fasting pH) and 12.00 h (postprandial pH) before feeding. After the measurements with an electronic pH meter (Seven Multi, Mettler-Toledo GmbH, Schwerzenbach, Switzerland), the urine was stored at −40°C before further analysis.

For the preparation of the urine analysis, the urine was defrosted at room temperature. For each cat, a collective sample was prepared and the total volume was measured. After thorough mixing, 12 ml of each collective sample were taken for the analysis, and the remaining urine was frozen at −80°C. Hydrochloric acid (37%) was added to the urine samples to adjust a pH of 2 (pH-meter Seven Multi, Mettler-Toledo GmbH). The amount of hydrochloric acid was documented. 300 µl of the acidified urine were stored at −80°C for the measurement of nitrogen. The remaining urine was filtered (Syringe Filter, Bulk, SFCA (surfactant-free cellulose acetate), 0.2 µm, 25 mm non-sterile, Thermo Scientific, Rochester, NY, USA) and stored at −80°C for the further analysis of urea, creatinine, and anions and cations.

### Urinary Nitrogen

The urine samples were defrosted and the concentrations of nitrogen were analyzed using the vario MAX CN Makro-Elementaranalysator (elementar Analysesysteme GmbH, Hanau, Germany).

### Urinary Creatinine and Urea

For the measurement of the concentrations of urinary urea, 500 µl of the urine samples and 500 µl of a buffer (1.25 g lithium carbonate, 2.5 g orthoboric acid, 246.25 ml ultra-pure water) were pipetted into high performance liquid chromatography (HPLC)-vials, locked with a PTFE/Silicone snap-cap (both Agilent Technologies, Waldbronn, Germany) and mixed (Vortex-Genie 2, Scientific Industries Inc., Bohemia, NY, USA).

For the measurement of urinary creatinine, 500 µl of the urine were diluted with 49.5 ml of ultra-pure water. One ml of this dilution was pipetted into glass vials and locked with a PTFE/Silicone snap-cap (both Agilent Technologies).

The concentrations of urea and creatinine were measured in the prepared samples using a HPLC method on an Agilent 1100 with UV-detector (Agilent Technologies). As a stationary phase, a reversed phase column (Phenomenex Synergi Hydro-RP, Phenomenex Ltd., Aschaffenburg, Germany) was used. The mobile phases were a phosphate buffer (0.1 M, potassium dihydrogen orthophosphate, ultra-pure water) and a mixture (50∶50) of methanol and ultra-pure water.

For calibration, a standard solution was used, with a total concentration of 100 µg urea/ml and 20 µg creatinine/ml.

The injection volume for each urine sample was 10 µl, and the flow rate 1 ml/min. The temperature of the column oven was 25°C, and the detection of urea and creatinine was performed at a wave length of 235 nm.

### Urinary Anions

For the measurement of the urinary anions (sulphate and phosphate as major anions; Ox and citrate as minor anions), an ion exchange chromatography system (Dionex DX-500) was used. A gradient pump (GP50), an electrochemical detector (ED40), an autosampler (ICS-3000 AS; all Dionex Corp., Sunnyvale, CA, USA) and a column cooler (IGLOO-CIL; Esslab, Hadleigh, Essex, United Kingdom) were included. The analytical column was a Dionex IonPac AS11-HC, the pre-column a Dionex IonPac AG11-HC, and the ion suppressor a Dionex SRS ULTRA II 4-mm (all Dionex Corp.).

The urine samples were defrosted and mixed in an ultrasonic bath. The samples were diluted (dilution factor for the major anions: 100; dilution factor for the minor anions: 10) and 1 ml of each dilution was pipetted into glass vials and locked with a PTFE/Silicone snap-cap (both Agilent Technologies).

For calibration, a standard solution, based on ultra-pure water, was used, with a total concentration of 250 µg chloride/ml, 20 µg sulphate/ml, 30 µg phosphate/ml, 10 µg Ox/ml and 10 µg citrate/ml. For the minor anions, four standard solutions, based on ultra-pure water, were prepared, with 50, 25, 10 and 1 µg Ox resp. citrate/ml.

For the analysis of the anions, the vials were stored in the autosampler at 10°C. 25 µl of each sample were injected into the system, the flow rate was 1 ml/min, and the analytical column had a temperature of 20°C.

### Urinary Cations

The urinary cations (sodium (Na), potassium (K) and ammonium (NH_4_) as major cations; Mg and Ca as minor cations) were detected using ion exchange chromatography (Dionex DX-120). An electrochemical detector and a cooled autosampler with an automatic dilution function (Dionex AS3500; all Dionex Corp.) were used.

The analytical column was a Dionex IonPac CS12A, the pre-column a Dionex IonPac CG12A and the ion suppressor a Dionex CSRS ULTRA II 4-mm (all Dionex Corp.).

The urine samples were defrosted and mixed thoroughly in an ultrasonic bath. The samples were diluted with a lithium standard buffer (500 µg lithium chloride/ml) and ultra-pure water. The dilution factor was 500 for the major cations and 10 for the minor cations. One ml of these dilutions was pipetted into plastic (major cations) resp. glass (minor cations) vials and locked with a PTFE/Silicone snap-cap (all Agilent Technologies).

For calibration, two lithium standard solutions (50 and 500 µg lithium chloride/ml) were produced first. For ammonium, four standards, based on hydrochloric acid (0.01 M) and the lithium standards were used, with a total concentration of 10, 6, 3 und 1 µg/ml ammonium and 5 µg/ml lithium chloride. For Na, K, Mg and Ca, a mixed standard solution with 5 µg/ml lithium chloride, 20 µg/ml Na, 10 µg/ml ammonium, 20 µg/ml K, 10 µg/ml Mg and 4 µg/ml Ca was used. The mixed standard solution was diluted with hydrochloric acid (0.01 M).

The vials were stored in the autosampler at 10°C. The samples were injected into the system with a volume of 25 µl, the flow rate was 1 ml/min, and the analytical column had room temperature.

### Analysis of the Data (Urine)

The analysis of the chromatograms of urea, creatinine, anions and cations was performed using Chromeleon Client software, version 6.80 SP2 (Dionex Corp.).

### Microscopic Evaluation of the Urine

The urine of the cats was microscopically evaluated for the formation of CaOx crystals (Zeiss Axiolab, Carl Zeiss Microscopy GmbH, Göttingen, Germany). Therefore, 5 ml of the urine were centrifuged at 2000×g and 4°C for 5 min (Heraeus Megafuge 1.0R, Thermo Scientific, Karlsruhe, Germany), and one drop of the urinary sediment was consecutively pipetted on a microscope slide.

### Preparation of the Faeces

The frozen faeces of the cats were weighed and lyophilized in a vacuum freeze-dryer (Lyovac GT2, LC Didactic, Hürth, Germany) over three days at the minimum or until weight constancy. In order to separate the plastic pellets, the samples were ground and the plastic pellets were removed manually before diminution (Mill: ZM 100, Kurt Retsch, Haan, Germany). The samples were ground to a particle size of 0.25 mm and stored in glass boxes until the following analysis.

### Minerals in the Faeces

One g of each freeze dried faecal sample was ashed for 12 h at 600°C. The ash was mixed with 6 ml hydrochloric acid (37%) and 20 ml distilled water in a beaker, and the beaker was covered and put into a warm sand bath for 50 min at 210–220°C. After cooling, the samples were transferred by an ash free filter into 50 ml graduated flasks, and the graduated flasks were filled up with distilled water to the measure mark. Subsequently, the mineral content in the samples was determined.

The phosphorus content was measured spectrophotometrically [22]. The extinctions were determined with an Ultrospec 2000 (Pharmacia Biotech, Cambridge, UK) at a wave length of 436 nm.

The concentrations of Ca, Na, K and Mg were measured using atomic absorption spectrometry. For this, a flame atomic absorption spectrometer (type vario 6) with an autosampler (AS 52) was used (Analytik Jena AG, Jena, Germany).

The apparent digestibility (AD) of the minerals was calculated as follows:
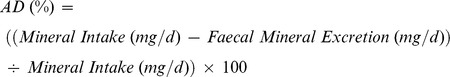



### Ox in the Feed and in the Faeces

The concentrations of Ox in the feed and faeces of the cats were measured using a commercial oxalate oxidase assay (Enzytec ™ Oxalsäure; # E2100; R-Biopharm AG, Darmstadt, Germany). One g of each sample was mixed with 4 ml hydrochloric acid (5 N) for 15 min (Multi Reax, Heidolph Instruments GmbH & Co. KG, Schwabach, Germany). Subsequently, the samples were heated at 60°C for 3 h (Haake C10-W13, Thermo Scientific). After centrifugation for 15 min at 2663×g and room temperature (Heraeus Labofuge 400R, Thermo Scientific), the aqueous phase was filtered (Acrodisc® Syringe Filter, 0.45 µm HT Tuffryn® Membrane, Pall Life Sciences, Port Washington, NY, USA) and an aliquot of 500 µl was adjusted to a pH of 2.9–3.1, either by adding hydrochloric acid or sodium hydroxide. The aliquots were centrifuged for 10 min at 17,000×g and room temperature (Heraeus Fresco 17 Centrifuge, Thermo Scientific). For the following measurement of Ox, the supernatant was used. Therefore, 10 µl of the sample and 200 µl of Reagent 1 (Buffer, Enzytec ™, R-Biopharm) were mixed on an orbital shaker (BioShake iQ, Analytic Jena) for 1 min at 1050 rpm and 37°C. Subsequently, the samples were incubated for 5 min (TECAN infinite M200 PRO, Tecan Group Ltd, Männedorf, Switzerland) at 37°C and 590 nm. Then, 20 µl of Reagent 2 (oxalate oxidase, Enzytec ™, R-Biopharm) were added and the samples were mixed on an orbital shaker (BioShake iQ, Analytic Jena) for 1 min at 1050 rpm and 37°C. Subsequently, the samples were incubated (TECAN infinite M200 PRO, Tecan Group Ltd) at 37°C and 590 nm. After 1 h, the extinctions were measured in the samples (TECAN infinite M200 PRO, Tecan Group Ltd). The oxalate concentrations were calculated as specified by the manufacturer (Enzytec ™, R-Biopharm). The apparent digestibility of Ox was calculated as described for the minerals.

### Blood Analysis

The blood samples were stored at room temperature for 1 hour before the following preparation. While the EDTA tubes were stored in the fridge at 4°C, the serum tubes were centrifuged at 4°C and 1811×g for 10 min (Heraeus Megafuge 1.0R, Thermo Scientific). Subsequently, the serum was pipetted into a 2 ml tube and stored in the fridge before all blood samples were sent to an external laboratory (Laboklin, Bad Kissingen, Germany; cooled transport).

The Ca and phosphate concentrations were photometrically detected, and the blood count as well as the measurement of urea and creatinine was performed using an automatic method. For the PTH measurement, a commercial iPTH sandwich ELISA for human diagnostics was used, which has been validated for cats in this laboratory.

### Statistical Analysis

The data were analyzed with SPSS 19 (SPSS Inc., Chicago, Illinois, USA). Kolmogorov-Smirnov- and Shapiro-Wilk-tests were performed to test for normal distribution. Normally distributed data were compared by one-factor analysis of variance (fixed factor diet) and Scheffé (variance equality) resp. Tamhane 2 (variance inequality) post hoc tests. Not normally distributed data were analyzed using the nonparametric Mann-Whitney-U-test. The data are presented in tables as means and standard deviation. Different letters in the same row indicate significant differences (p≤0.05) among the groups.

## Results

### Animal Health, BW, Feed and Water Intake and Urine Volume

The cats were healthy during the whole study and no effect of the diets on the BW, feed intake or urine volume was observed (Table 2). The daily water intake was generally low, with a higher consumption after feeding the diet B compared to the diet C.

**Table 2:** Body weight (BW), feed and water intake, urine volume, urinary pH and urine composition of the cats fed a diet with 12.2 (A), 18.5 (B) and 27.0 (C) g Ca/kg DM.

	Diet A	Diet B	Diet C
* 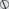 BW (kg)	4.43±1.39	4.43±1.31	4.35±1.26
Feed intake(g/kg BW/d)	65.5±14.7	59.6±15.2	64.9±13.6
Water intake(g/kg BW/d)	5.04±2.85^ab^	5.08±2.15^a^	4.65±3.39^b^
* Urine volume(ml/kg BW/d)	36.2±11.7	29.5±8.92	34.7±8.02
**Urinary**			
* Fasting pH	6.60±0.10	6.69±0.17	6.78±0.20
Postprandial pH	6.76±0.23^ab^	6.60±0.38^a^	6.91±0.27^b^
Ca (mg/l)	35.3±17.2	33.9±6.29	29.7±8.57
* P (mg/l)	1750±249	1821±226	1699±175
* Mg (mg/l)	29.2±9.52	31.6±9.78	29.6±5.60
K (mg/l)	2716±454^a^	3279±712^b^	3250±297^b^
* Na (mg/l)	2279±335^a^	2814±355^b^	2785±208^b^
* Urea (mg/l)	25.4±5.39	26.7±3.14	34.4±9.31
Creatinine (mg/l)	1006±229	1403±472	1166±194
Sulphate (mg/l)	1587±176^a^	1628±212^ab^	1415±105^b^
* Ox (mg/l)	29.7±12.3	28.4±6.15	27.5±5.84
* Citrate (mg/l)	781±172^a^	924±132^ab^	1048±193^b^
* Ammonium(mg/l)	1406±169	1477±197	1323±155
Nitrogen (g/l)	17.4±2.63^ac^	20.7±2.80^b^	18.2±1.20^c^

n = 8/diet; mean ± standard deviation.

* normally distributed data.

Mean values within a row with different superscript letters differ (*P*≤0.05).

### Urinary pH, Urine Composition and Microscopic Characterization of the Urine

The fasting urinary pH, measured at 7.00 h in the morning, did not differ depending on the experimental diets (Table 2). The measurement of the postprandial urinary pH (12.00 h) demonstrated that the pH was the lowest, when the cats were fed the diet B (6.60) and the highest after feeding diet C (6.91).

The urinary concentrations of Ca were between 29.7 to 35.3 mg/l and were not influenced by the varying Ca and P intake (Table 2). The different diets had also no impact on the concentration of urinary P, which ranged from 1699 mg/l to 1821 mg/l. The urinary concentration of Mg was also largely independent of the dietary Ca and P levels and ranged around 30 mg/l.

The urinary Na concentrations were lower, when the cats were fed the diet with the lowest Ca concentration (2279 mg/l) compared to the diets with the higher Ca concentrations, where the urinary Na concentrations ranged between 2785 and 2814 mg/l. Urinary K was only slightly affected by the diets, with the lowest concentrations after feeding diet A compared to the diets B and C (*P*≤0.05). The concentrations of urea, creatinine, Ox and ammonium were similar and seemed independent of the diets. The sulphate concentrations in the urine were only marginally affected, with a small group difference between diet A and C. Interestingly, the urinary citrate concentrations increased with higher levels of Ca in the diets, from 781 mg/l in period A to 924 and 1048 mg/l in periods B and C (*P*≤0.05). The concentration of nitrogen in the urine was increased in the experimental period B compared to A and C (*P*≤0.05).

As with the mostly unaffected urinary mineral concentrations, the urinary mineral excretion was also not influenced by the diets (Table 3). Because of increasing Ca levels in the diets A, B and C, the ratio between the Ca excretion and the Ca intake was higher (1.00%) when the cats were fed the diet A compared to the diets B (0.51%) and C (0.34%). Considering that not only Ca, but also P increased in the diets, the same observation applied for the ratio between the P excretion and the P intake, with higher values in period A (36.5%) compared to the periods B (29.1%) and C (24.5%).

**Table 3:** Urinary mineral and Ox excretion of the cats fed a diet with 12.2 (A), 18.5 (B) and 27.0 (C) g Ca/kg DM.

	Diet A	Diet B	Diet C
Urinary Ca excretion(mg/kg BW/d)	1.33±1.03	0.99±0.31	1.06±0.51
Urinary Ca excretion/Caintake (%)	1.00±0.62^a^	0.51±0.07^b^	0.34±0.11^c^
* Urinary P excretion(mg/kg BW/d)	61.0±12.1	52.6±12.0	57.9±9.38
* Urinary P excretion/Pintake (%)	36.5±7.40^ac^	29.1±3.37^c^	24.5±1.58^b^
* Urinary Na excretion(mg/kg BW/d)	79.3±15.1	82.2±22.7	97.1±25.5
* Urinary Na excretion/Naintake (%)	78.7±16.2	81.9±11.0	79.8±9.03
* Urinary Mg excretion(mg/kg BW/d)	0.99±0.23	0.89±0.23	1.03±0.34
* Urinary Mg excretion/Mgintake (%)	10.6±2.91	9.64±3.37	8.08±1.57
* Urinary K excretion(mg/kg BW/d)	95.7±24.8	93.9±22.6	113±29.2
* Urinary K excretion/Kintake (%)	79.3±17.3	89.1±14.5	94.8±10.8
* Urinary Ox excretion(mg/kg BW/d)	0.98±0.23	0.82±0.22	0.93±0.19
Urinary Ox excretion/Oxintake (%)	125±40.1^a^	58.0±11.6^b^	148±24.6^a^

n = 8/diet; mean ± standard deviation. BW: body weight.

* normally distributed data.

Mean values within a row with different superscript letters differ (*P*≤0.05).

As the Ox concentrations were the highest in the diet B compared to the other diets, the ratio between the daily renal Ox excretion and the Ox intake was the lowest after feeding diet B (58.0%) compared to the diets A (125%) and C (148%).

No CaOx crystals were microscopically detected in the urine of the cats during the whole study.

### Faecal DM, Mineral and Ox Concentrations in the Faeces and Apparent Digestibility of the Diets

The faecal DM ranged between 40.8% and 41.8% and did not differ between the experimental groups (Table 4).

**Table 4:** Dry matter (DM) of the faeces, daily amount of faeces, faecal mineral and Ox excretion and apparent digestibility of the minerals and Ox in cats fed a diet with 12.2 (A), 18.5 (B) and 27.0 (C) g Ca/kg DM.

	Diet A	Diet B	Diet C
* DM of the faeces (%)	40.9±3.93	41.8±3.77	40.8±3.97
* Amount of faeces(g DM/d)	8.08±1.33^ab^	7.33±2.03^a^	9.61±1.57^b^
Faecal Ca (mg/g DM)	92.4±7.64^a^	106±8.13^b^	132±3.93^c^
* Faecal Ca excretion(mg/d)	740±86.7^a^	777±224^a^	1264±195^b^
* Faecal Ca excretion(mg/kg BW/d)	181±52.5^a^	186±66.0^a^	303±56.2^b^
Apparent digestibilityof Ca (%)	−36.9±15.9^a^	4.33±16.5^b^	−0.24±10.2^b^
* Faecal P (mg/g DM)	40.4±4.76^a^	45.5±1.99^a^	61.9±5.20^b^
* Faecal P excretion(mg/d)	323±37.3^a^	335±101^a^	595±109^b^
* Faecal P excretion(mg/kg BW/d)	79.0±22.6^a^	80.6±31.1^a^	141±21.5^b^
Apparent digestibilityof P (%)	54.5±5.82^a^	56.7±8.19^a^	39.6±9.05^b^
* Faecal Na (mg/g DM)	4.42±1.27	3.56±0.74	3.94±0.55
* Faecal Na excretion(mg/d)	35.2±9.86^ab^	26.0±8.01^a^	37.9±8.45^b^
* Faecal Na excretion(mg/kg BW/d)	8.86±3.98	6.32±2.83	9.02±1.89
* Apparent digestibilityof Na (%)	91.8±2.45	93.9±1.56	92.4±1.21
* Faecal Mg (mg/g DM)	4.22±0.31	3.97±0.16	4.19±0.28
Faecal Mg excretion(mg/d)	34.0±5.60^a^	29.0±7.55^a^	40.2±6.87^b^
Faecal Mg excretion(mg/kg BW/d)	8.18±2.02^ab^	6.95±2.45^a^	9.61±1.74^b^
* Apparent digestibilityof Mg (%)	15.0±9.84^a^	28.8±11.4^b^	23.4±6.47^ab^
Faecal K (mg/g DM)	2.44±1.23	2.39±0.92	2.22±0.51
Faecal K excretion(mg/d)	20.0±11.1	17.5±8.29	21.0±4.79
Faecal K excretion(mg/kg BW/d)	5.10±3.83	4.49±3.19	5.16±1.84
Apparent digestibilityof K (%)	96.1±2.23	96.1±1.77	95.7±0.81
Faecal Ox (mg/g DM)	0.04±0.01^a^	0.03±0.00^b^	0.03±0.01^b^
* Faecal Ox excretion(mg/d)	0.36±0.11	0.25±0.08	0.29±0.08
Faecal Ox excretion(mg/kg BW/d)	0.08±0.03^a^	0.06±0.02^b^	0.07±0.02^ab^
Apparent digestibilityof Ox (%)	89.4±2.98^a^	95.9±1.00^b^	88.9±2.84^a^

n = 8/diet; mean ± standard deviation. BW: Body weight.

* normally distributed data.

Mean values within a row with different superscript letters differ (*P*≤0.05).

The mineral concentrations in the faeces of the cats were markedly affected by the diets. With increasing dietary Ca, the faecal Ca concentrations increased from 92.4 mg/g DM to 132 mg/g DM. Moreover, the faecal Ca excretion was almost 60% higher after feeding diet C compared to the diet A.

Similar to the faecal Ca excretion, the P excretion was also affected by the diets. The diet with the highest Ca and P levels (diet C) resulted in the highest P concentrations in the faeces (61.9 mg/g DM compared to 40.4 mg/g DM) and therefore in an approximately 55% higher faecal P excretion compared to the feeding of the diet A.

The faecal concentrations of Na, K and Mg did not differ among the feeding groups, and no or only small effects were observed for the excretion of these minerals. The faecal Mg excretion (mg/d) was the highest after feeding diet C compared to the diets A and B (*P*≤0.05). Referred to the BW (mg/kg BW/d), the faecal Mg excretion was only higher after feeding the diet C compared to the diet B.

Feeding the diet A induced slightly higher faecal Ox concentrations (0.04 mg/g DM) than feeding the diets B and C (both 0.03 mg/g DM) (*P*≤0.05). The daily faecal Ox excretion was also only marginally affected by the diets, with the lowest excretion in group B (0.06 mg/kg BW/d) compared to group A (0.08 mg/kg BW/d) (*P*≤0.05).

The apparent digestibility of the minerals and Ox is also summarized in Table 4. Ca showed variable values, with −36.9% in period A, and 4.33% and −0.24% in the periods B and C. The apparent P digestibility was 54.5% and 56.7% in the periods A and B compared to 39.6% in period C (*P*≤0.05). The apparent digestibility of Na was high (91.8–93.9%) without diet related effects of the diets (*P*>0.05). The apparent digestibility of Mg was with 15.0% the lowest after feeding diet A, and increased after feeding the diets B and C to 28.8% and 23.4%. K also achieved relatively high apparent digestibilities, the range was quite narrow by 96%, with no significant differences between the feeding periods.

The apparent Ox digestibility ranged between 88.9% and 95.9% and was the highest after feeding the diet B compared to the diets A and C (*P*≤0.05).

### Blood Parameters

No group differences were observed in the blood counts of the cats (data not shown) and all parameters were within the normal range for cats. Increasing dietary Ca levels resulted in a moderate decrease of the serum Ca levels, but a marked decrease of the serum PTH levels of the cats (Table 5). The P concentrations in the blood were not affected by the diets.

**Table 5:** Urea, creatinine, Ca, phosphate and PTH in the blood of cats fed a diet with 12.2 (A), 18.5 (B) and 27.0 (C) g Ca/kg DM.

	Diet A	Diet B	Diet C	Reference range^1^
* Urea (mmol/l)	8.19±0.80^a^	9.83±0.80^b^	9.33±0.66^b^	5.00–11.3
* Creatinine (µmol/l)	139±34.9	157±27.4	150±32.3	0.00–168
Ca (mmol/l)	2.63±0.09^a^	2.59±0.04^ab^	2.50±0.12^b^	2.30–3.00
Phosphate, anorg. (mmol/l)	1.39±0.16	1.49±0.13	1.40±0.17	0.80–1.90
PTH (pg/ml)	21.9±7.26^a^	6.68±7.12^b^	4.41±2.94^b^	3.30–22.5

n = 8/diet; mean ± standard deviation.

* normally distributed data;

1 Laboklin (Bad Kissingen, Germany).

Mean values within a row with different superscript letters differ (*P*≤0.05).

## Discussion

Considering the varying Ca concentrations in commercial diets, the daily Ca and P intake of cats can be far of the recommended requirement. For adult cats, the recommended allowance is 2.9 g Ca/kg DM and 2.6 g P/kg DM (with 16.8 MJ ME/kg DM) [21]. A high dietary Ca intake is considered as a risk factor for the formation of urinary crystals and uroliths, especially for CaOx stones, because of an increased urinary Ca excretion and the impact on the urinary saturation. This study demonstrated that increasing dietary Ca and P by adding bone meal to a typical canned diet did not affect the urinary mineral concentrations, but had a major influence on the PTH levels and a moderate impact on the Ca levels in the blood of cats. Moreover, an increase of the faecal Ca was observed. For that reason, homeostatic regulatory processes of the Ca metabolism in cats seem to be related to the intestinal tract and not to the renal excretion.

The regulation of the Ca and P homeostasis in monogastrics is mainly mediated by PTH and calcitriol [23]–[24]. In monogastric animals, PTH stimulates the renal Ca absorption and phosphate excretion, and activates the 25-hydroxyvitamin D-1α-hydroxylase, which is localized in the kidneys and serves for an increased synthesis of calcitriol [25]–[26]. Moreover, PTH inhibits the renal 24-hydroxylase, which is mainly responsible for an increased degradation of calcitriol [27]. PTH induces a release of Ca and phosphate in the bones [28], however, it could also have anabolic effects on bones [29]. In addition to PTH, calcitriol also enhances the blood Ca concentration by increasing the intestinal Ca absorption, reducing the renal Ca excretion and increasing the Ca mobilization from the skeleton [30]–[32]. Since the present study focused on the impact of dietary Ca on the PTH levels in the blood of cats, future studies should also evaluate the potential affection of the feline calcitriol secretion.

In the present study, three experimental diets with increasing levels of both, Ca and P, were evaluated. The reason for the increasing Ca and P levels can be found in the dietary Ca source used in the present study, which was bone meal, containing mainly Ca apatite as the crystallized form of Ca phosphate. Therefore, increasing dietary Ca levels were accompanied by increasing dietary P levels. The increasing Ca levels in the diets did not result in a hypercalcaemia or an enhanced renal Ca excretion. Instead, the Ca concentration in the blood was slightly decreased and the urinary Ca concentrations were unaffected. Therefore, effective regulatory processes of the organism can be assumed, which is strongly supported by the measured decreased PTH levels in the blood and the increased Ca concentrations in the faeces when the cats were fed the high Ca diets. A higher provision of Ca with the diet has obviously resulted in a lower synthesis and secretion of PTH compared to the situation of a lower Ca intake with diet A. However, all values for PTH were within the normal range of 3.3–22.5 pg/ml given by the analyzing laboratory, which was nearly similar to reference data given in the literature [33].

The increasing dietary P levels in the experimental diets seemed to have not affected the PTH secretion. In general, high serum phosphate concentrations result in a reduced activation of calcitriol in the kidneys, a reduced mobilization of Ca and phosphate in the bones, and a reduced intestinal absorption of Ca and phosphate. Therefore, the Ca concentrations in the blood decrease, which results in an increased synthesis and secretion of PTH in the parathyroid glands. The increased PTH levels then lead to an increased renal P excretion and therefore a reduction of the P levels in the blood [34]. In addition to these described relations between Ca, P and PTH, the Ca:P-ratio of the diets is an important factor influencing the intestinal P absorption and therefore the Ca and P status in cats. The optimum Ca:P-ratio for cats was recommended to be close to 1 (between 0.9–1.1∶1) [35], however, there is some evidence that Ca:P-ratios below 1 could be critical for the renal function [36]. Moreover, a wide Ca:P-ratio decreases the intestinal P absorption, which has already been demonstrated [36]. In contrast, no effect of increasing dietary P, accompanied by unchanged dietary Ca, was observed for the intestinal Ca absorption, renal Ca excretion or for the serum Ca concentrations in cats [37].

In the present study, two factors, the increased Ca and the increased P in the experimental diets may have influenced the PTH secretion. While increasing dietary Ca levels should lead to a decreased PTH secretion, the higher P levels in the diets would generally stimulate the PTH secretion. It can be therefore hypothesized that dietary Ca had a stronger impact on PTH compared to P, because of the observed decrease of the serum PTH concentrations with increasing levels of dietary Ca and P. The Ca:P-ratios differed among the experimental diets, but were not too far from the recommendations [35]. It can therefore be assumed that the intestinal availability of Ca or P was not vitally affected by the dietary Ca:P-ratio. It can be suggested that the observed modulatory processes within the hormonal balance could be a reason for the unaffected urinary mineral concentrations in the present study. The affected PTH secretion may have especially regulated the intestinal absorption of the minerals and therefore no differences in the urinary mineral excretion depending on the diets could be observed.

The data particularly demonstrate that the urinary Ca and Ox excretion was independent of the daily Ca intake. Therefore, the present study can not support the hypothesis [2]–[3] that high dietary Ca levels cause an intestinal complexation of Ca with Ox and therefore a reduced renal Ca and Ox excretion. In this context, it seems to be important that cat food normally contains only small amounts of Ox, which was affirmed by the present study (0.06–0.14 g Ox/kg DM). Therefore, the faecal Ox concentrations and daily faecal Ox excretion were only slightly affected by the diets. The ratio between the renal Ox excretion and the Ox intake suggests that endogenous Ox is of major importance in cats. However, considering the low levels of dietary Ox, an impact on the intestinal complex formation with dietary Ca seems to be insignificant. This circumstance may be an explanation for the divergent present results compared to the related data from studies in humans and dogs [2]–[3].

In conclusion, the present data demonstrated that varying Ca apatite levels in a canned food did not influence the urine composition in healthy adult cats and may therefore be no risk factor for the formation of CaOx uroliths. This is also supported by the data on the microscopic evaluation of the urine, as no CaOx crystals were detected during the whole study. However, there are some limiting factors of the study which should be considered for the interpretation of the present results:

The experimental diets used in the present study were canned diets with a moisture content of 83.7% (diet A) and 82.6% (diets B and C). It has been recently demonstrated that a high water intake markedly influenced urinary parameters in cats, especially by increasing the urine volume, reducing the specific gravity of the urine and decreasing the urinary relative supersaturation of CaOx [38]. Therefore, the present results may differ in the case of a dry diet with increasing levels of Ca apatite.

A further limiting factor could be the age of the cats used in the present study. It has been reported that feline CaOx uroliths were found in all age groups, however, most cases appeared when the cats were over 4 years old [39]. Another study demonstrated that nearly 25% of the affected cats with CaOx were between 3 and 5 years old, but approximately 70% were over 5 years old [1]. Based on these data, it should be taken into consideration that our cats were 36±4 months old and therefore not in the highest, but in a lower risk group for the development of CaOx uroliths. Moreover, the animal number in the present study was relatively low, which generally limits the significance of the results.

In addition to the unaffected renal Ca excretion, the diets also did not influence the urinary concentrations of other minerals. In particular, some authors supposed an influence on the renal P and Mg excretion [6], [9]. Therefore, varying levels of dietary Ca apatite seem to be also no risk factor for the formation of other uroliths like MAP or Ca phosphate.

The urinary pH was only slightly affected by increasing levels of Ca apatite in the diets, and no strong acidifying or alkalizing effect, as provided by dietary Ca chloride or Ca carbonate, was observed. However, the pH-measurements demonstrated that the diet with the highest Ca and P concentration (diet C) resulted in higher pH values (fasting/postprandial: 6.78/6.91) compared to the diets with lower Ca concentrations (diet A: 6.60/6.76; diet B: 6.69/6.60). In general, the urinary pH ranged between 6.60 and 6.91. These values may not be relevant for the formation of CaOx or MAP uroliths. While an acidification of the urine is considered for the treatment of MAP, such low pH values, especially a urinary pH <6.29, are also a risk factor for the formation of CaOx uroliths [12], [40]. In contrast, MAP can efficiently precipitate when the urinary pH is >7 [5]. Although the present diets with low and higher Ca apatite levels resulted in an almost similar urinary pH, which could not be considered as a specific risk factor for the formation of uroliths, it should be noted that in the case of existing uroliths or predisposed cats, specific diets with acidifying or alkalizing components should be used.

With regard to the discussed effects of the diets on the urinary pH, the data on the citrate concentrations in the urine of the cats should be considered. Citrate is a crystallisation inhibitor for Ca phosphate and CaOx uroliths [41]–[42] and can be provided with the diets or renally synthesised. As the citrate concentrations in diets for cats are low, it can be hypothesized that the observed increase of urinary citrate in the group receiving the high Ca diet resulted from an increased citrate synthesis and excretion by the kidneys. Taking into consideration that a low urinary pH inhibits and a higher urinary pH stimulates the urinary citrate excretion [42], the small increase of the urinary pH observed with the high Ca diet could be an explanation for the higher urinary citrate concentrations in this group.

In conclusion, the present data demonstrated that the urinary Ca excretion in healthy cats was independent of the dietary concentrations of Ca apatite when added to a typical canned diet over a relatively short time period. The diets had a strong effect on the PTH levels in the blood of the cats, and modulatory processes within the Ca balance can be assumed. The results may differ in the case of alterations in the study design, especially by using a dry feed or cats with a predisposition for or existing feline uroliths.

## Supporting Information

Table S1 **Urinary pH and urine composition of the cats fed a standard diet^1^ before the beginning of the present study.** n = 8/diet; mean ± standard deviation.
(DOCX)
