# Supplementary material for: Urinary Calcium and Oxalate Excretion in Healthy Adult Cats Are Not Affected by Increasing Dietary Levels of Bone Meal in a Canned Diet
Source: PLoS One. 2013 Aug 5;8(8):e70530. doi: 10.1371/journal.pone.0070530 (PMC3734279; doi:10.1371/journal.pone.0070530)
Supplement: Table S1 — Urinary pH and urine composition of the cats fed a standard diet1 before the beginning of the present study. n = 8/diet; mean ± standard deviation. (DOCX) [file pone.0070530.s001.docx]

**Table S1:** Urinary pH and urine composition of the cats fed a standard diet^1^ before the beginning of the present study. n = 8 / diet; mean ± standard deviation.

|  | **Standard Diet** | | |
| --- | --- | --- | --- |
| Fasting pH | 7.12 | ± | 0.65 |
| Postprandial pH | 6.83 | ± | 0.84 |
| Ca (mg/l) | 51.8 | ± | 22.3 |
| P (mg/l) | 939 | ± | 270 |
| Mg (mg/l) | 137 | ± | 39.9 |
| K (mg/l) | 5140 | ± | 1011 |
| Na (mg/l) | 9198 | ± | 2348 |
| Urea (mg/l) | 60.5 | ± | 30.1 |
| Creatinine (mg/l) | 2034 | ± | 517 |
| Sulphate (mg/l) | 4048 | ± | 923 |
| Ox (mg/l) | 148 | ± | 38.7 |
| Citrate (mg/l) | 79.7 | ± | 66.2 |
| Ammonium (mg/l) | 1885 | ± | 462 |
| Nitrogen (g/l) | 30.3 | ± | 5.72 |

^1^Analyzed composition: Dry matter (DM): 929 g/kg, Crude protein: 365 g/kg DM, Crude fat: 105 g/kg DM, Crude fibre: 33.2 g/kg DM, Crude ash: 83.6 g/kg DM, Ca: 13.7 g/kg DM, P: 8.61 g/kg DM, Na: 5.36 g/kg DM, K: 6.35 g/kg DM, Mg: 0.95 g/kg DM; Calculated ME: 16.2 MJ/kg DM; Ingredients (according to the manufacturer): cereals, meat and animal by-products, vegetable protein extracts, oil, fat, vegetable by-products, minerals, vegetables
